# Potent sialic acid inhibitors that target influenza A virus hemagglutinin

**DOI:** 10.1038/s41598-021-87845-0

**Published:** 2021-04-21

**Authors:** Yu-Jen Chang, Cheng-Yun Yeh, Ju-Chien Cheng, Yu-Qi Huang, Kai-Cheng Hsu, Yu-Feng Lin, Chih-Hao Lu

**Affiliations:** 1 The Ph.D. Program of Biotechnology and Biomedical Industry, China Medical University, Taichung, Taiwan; 2 Department of Medical Laboratory Science and Biotechnology, China Medical University, Taichung, Taiwan; 3 Graduate Institute of Cancer Biology and Drug Discovery, Taipei Medical University, Taipei, Taiwan; 4 Department of Medical Laboratory Science and Biotechnology, Asia University, Taichung, Taiwan; 5 Graduate Institute of Biomedical Sciences, China Medical University, Taichung, Taiwan

**Keywords:** Computational biology and bioinformatics, Drug discovery

## Abstract

Eradicating influenza A virus (IAV) is difficult, due to its genetic drift and reassortment ability. As the infectious cycle is initiated by the influenza glycoprotein, hemagglutinin (HA), which mediates the binding of virions to terminal sialic acids moieties, HA is a tempting target of anti-influenza inhibitors. However, the complexity of the HA structure has prevented delineation of the structural characterization of the HA protein–ligand complex. Our computational strategy efficiently analyzed > 200,000 records of compounds held in the United States National Cancer Institute (NCI) database and identified potential HA inhibitors, by modeling the sialic acid (SA) receptor binding site (RBS) for the HA structure. Our modeling revealed that compound NSC85561 showed significant antiviral activity against the IAV H1N1 strain with EC_50_ values ranging from 2.31 to 2.53 µM and negligible cytotoxicity (CC_50_ > 700 µM). Using the NSC85561 compound as the template to generate 12 derivatives, robust bioassay results revealed the strongest antiviral efficacies with NSC47715 and NSC7223. Virtual screening clearly identified three SA receptor binding site inhibitors that were successfully validated in experimental data. Thus, our computational strategy has identified SA receptor binding site inhibitors against HA that show IAV-associated antiviral activity.

## Introduction

Seasonal influenza virus poses a severe threat to human health. An estimated 56 million people worldwide were infected with influenza virus between October 1, 2019 and April 4, 2020, causing serious illness in 39 to 56 million and 62,000 deaths^1^. Influenza-related deaths most commonly occur in people aged 65 years and over in industrialized countries^2^, while among young children, a systematic review published in 2008 of the global burden of respiratory infections related to seasonal influenza among young children calculated that of all deaths in children aged younger than five years attributable to influenza-associated acute lower respiratory infections, 99% occurred in developing countries^3^. The effectiveness of seasonal vaccines intended to protect against infection frequently fail, because of influenza's genetic drift and reassortment ability^4^. Yearly vaccination is the primary means of preventing and controlling influenza^5^, but vaccine efficacy varies according to genetic relatedness among viruses in the vaccine and circulating strains^6^. Currently, three influenza antiviral drugs approved by the US Food and Drug Administration (FDA) are recommended by the US Centers for Disease Control and Prevention (CDC) for use against circulating influenza viruses; Rapivab (peramivir), Relenza (zanamivir), and Tamiflu (oseltamivir), all of which are neuraminidase (NA) inhibitors and interfere with the release of the progeny virion from infected host cells. This process prevents the infection of new host cells and halts infection spread in the respiratory tract^7^. However, despite the apparent treatment efficacy shown by these NA inhibitors, they have also been associated with drug resistance. In Japan, oseltamivir‐resistant H1N1 viruses were isolated from 7 of 43 patients (16%) in 2000/2001^8^ and almost one-third (27%) of patients infected with H1N1 viruses between 2005 and 2007 exhibited resistance to oseltamivir in the UK^9^. We therefore sought to determine the feasibility of targeting the influenza virus glycoprotein hemagglutinin (HA) as a potential alternative therapeutic option to NA-mediated therapeutic strategies, capable of inhibiting influenza virus entry.

HA plays a key role during the influenza A virus (IAV) life cycle, particularly in the attachment and penetration of influenza virus strains to host cell molecules^10^. No HA inhibitors are as yet available for clinical use, mainly because of the diversity of HAs created by antigenic drift and shift^11,12^. One report has suggested that glycosylations in the globular head of the HA is an essential step for viruses to gain virulence and antigenic properties^13^. Mature HA is a homotrimer, with a globular domain containing a RBS specific for SA expressed by host cell glycoproteins and glycolipids^14^. When the RBS of influenza binds to the host SA, the viruses can quickly enter the host cells. To prevent IAV from obtaining entry to the host cells, the RBS might be a potential target.

The homotrimer is constructed by uncleaved HA0 subunits, which are unable to fuse the host membranes. By interrupting the correct folding of HAs in progeny influenza viruses, HA0 inhibitors elicit nonfunctional HA conformation and thus block viral entry^15^. For instance, the HA0 inhibitor AF4H1K1 blocks immature HA0 cleavage and inhibits the infectivity of IAV^16^. Another example is nafamostat, a serine protease inhibitor, which reduces cleavage of the precursor protein HA0^17^. By folding into a jelly-roll motif of eight stranded antiparallel β-sheets with a shallow pocket at the distal tip, the HA1 subunit acts as an RBS surrounded by antigenic sites^18^. The antiviral mechanism of HA1 inhibitors is mainly attributed to their ability to block receptor binding and prevent the acquisition of a viral infection. An analysis of extracts from a traditional medicinal plant used in Borneo for treating symptoms of influenza infection has revealed that the useful substances were not limited to SA-like compounds, but also included non-SA-like components that might act against other viral proteins besides HA and NA^19^. Other research has shown that synthetic SA-mimic peptides have a high affinity to the RBS and thus block the initial infection^20^. Another study has described how multivalent 6′-sialyllactose-polyamidoamine (6SL–PAMAM) conjugates, which include a dendrimer scaffold against the SA receptor binding site, are capable of effectively inhibiting IAV infection^21^. Notably, recent research has detailed the isolation and characterization of 1428A33/1, 1428B5/1 and F3A1, human monoclonal antibodies (mAbs) that target residues of the RBS of HA and neutralize A(H1N1)pdm09 escape mutant viruses that very occasionally escape from these mAbs^22^. The HA2 subunit mediates the host endosomal membrane's fusion with the viral membrane, allowing viral ribonucleoprotein entry into the host cell^23^. During the process of HA-mediated fusion, metastable HA2 subunits undergo irreversible rearrangement under acidic pH, which causes membrane fusion and the completion of viral entry^15^. The HA2 inhibitor Arbidol (umifenovir) interacts directly with HA2 and can prevent the acidification-induced conformation change of HA2, impeding fusion with endosome membranes^24^.

After considering all HA subunits in the IAV structure, our study focused on the RBS of the HA1 subunit. We analyzed records of compounds held in the United States National Cancer Institute (NCI) database to identify potential inhibitors. Over 200,000 compounds were screened using the molecular docking method. Docked compounds were used to recognize interaction preferences by analyzing the RBS with interacting residues and specific physical–chemical properties. These models enabled us to identify inhibitors with novel scaffolds. Several HA inhibitors were identified and validated by a series of bioassays, including cell proliferation, plaque reduction, and hemagglutination inhibition. To refine our analysis and discover more compounds targeting HA1, we subjected the initial compound's derivatives to the same experimental tests, which revealed some attractive leads for potential HA1 inhibitors. Our study describes our use of a comprehensive framework for efficient screening of lead compounds for further drug design and development as HA1 inhibitors.

## Results

### A framework of anchor construction and post-screening analysis

**Figure 1:**
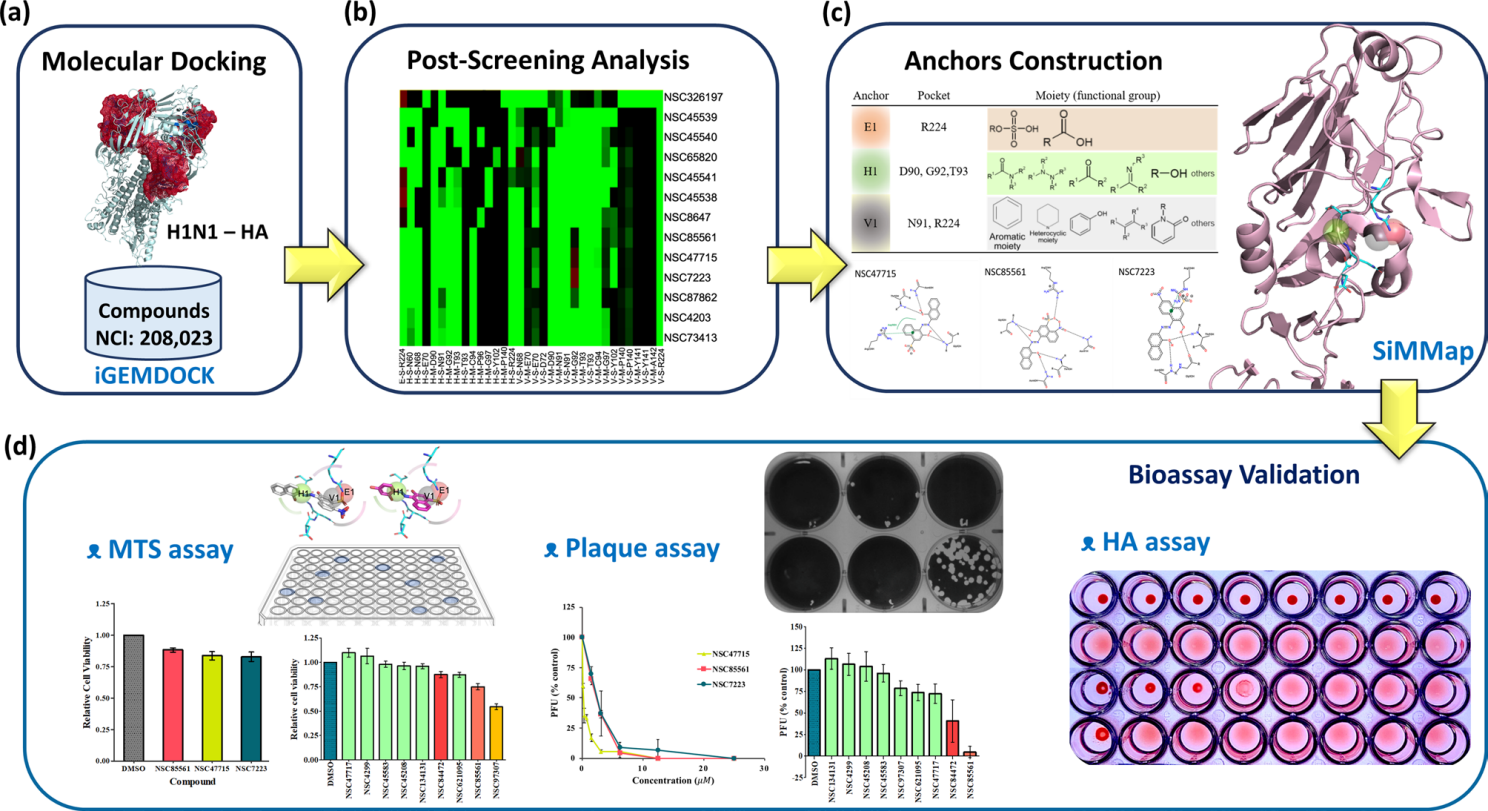
Framework of the study. **(a)** Virtual screening for HA inhibitors using docking compounds from the NCI and the H1N1 hemagglutinin (PDB ID: 1RUY) was based on the iGEMDOCK program. **(b)** Post-screening analysis was conducted by generating the consensus interaction profile between the docked compounds and HA residues. **(c)** Anchors were constructed using the SiMMap to detect the most favorable binding pockets and moieties. **(d)** Validation of potential compounds was performed using three bioassays: The MTS assay evaluated the toxicities of the compounds; the plaque reduction assay quantified the antiviral activity of the compounds versus IAV; and the hemagglutination inhibition assay measured influenza-specific levels in type-O human serum.

Figure 1 gives an overview of how we identified novel HA inhibitors by anchor construction and post-screening analysis. First, the structure of the RBS of the HA1 subunit (PDB ID: 1RUY)^25^ was selected as the target protein, and 208,023 NCI compounds were collected as the screening compound database (Fig. 1a). The molecular docking and post-screening analyses were performed by iGEMDOCK^26^ (Fig. 1b). Sialic acid served as the positive control and was docked into our prepared sialic acid RBS to validate our docking tool. SiMMap^27^ anchors were subsequently constructed from the SiMMap server (a site-moiety map for drug discovery and mechanisms server), based on the docking scores and the types of interactions between the 1,000 top-ranked compounds and the RBS (Fig. 1c). An anchor is composed of a binding pocket with corresponding interacting residues, moiety preference, and interaction type (E: electrostatic, H: hydrogen-bonding, or V: van der Waals forces). These 1,000 compounds were re-ranked based on the SiMMap score, and the top 20 compounds were selected as potential candidates (shown in Supplementary Table S1 online). Finally, nine compounds were purchased and evaluated by cell proliferation, plaque reduction, and hemagglutination inhibition assays in vitro (Fig. 1d). Figure 1 illustrates the process of how we conducted our research and validated our hypothesis.

### Anchors of the receptor-binding site

**Figure 2:**
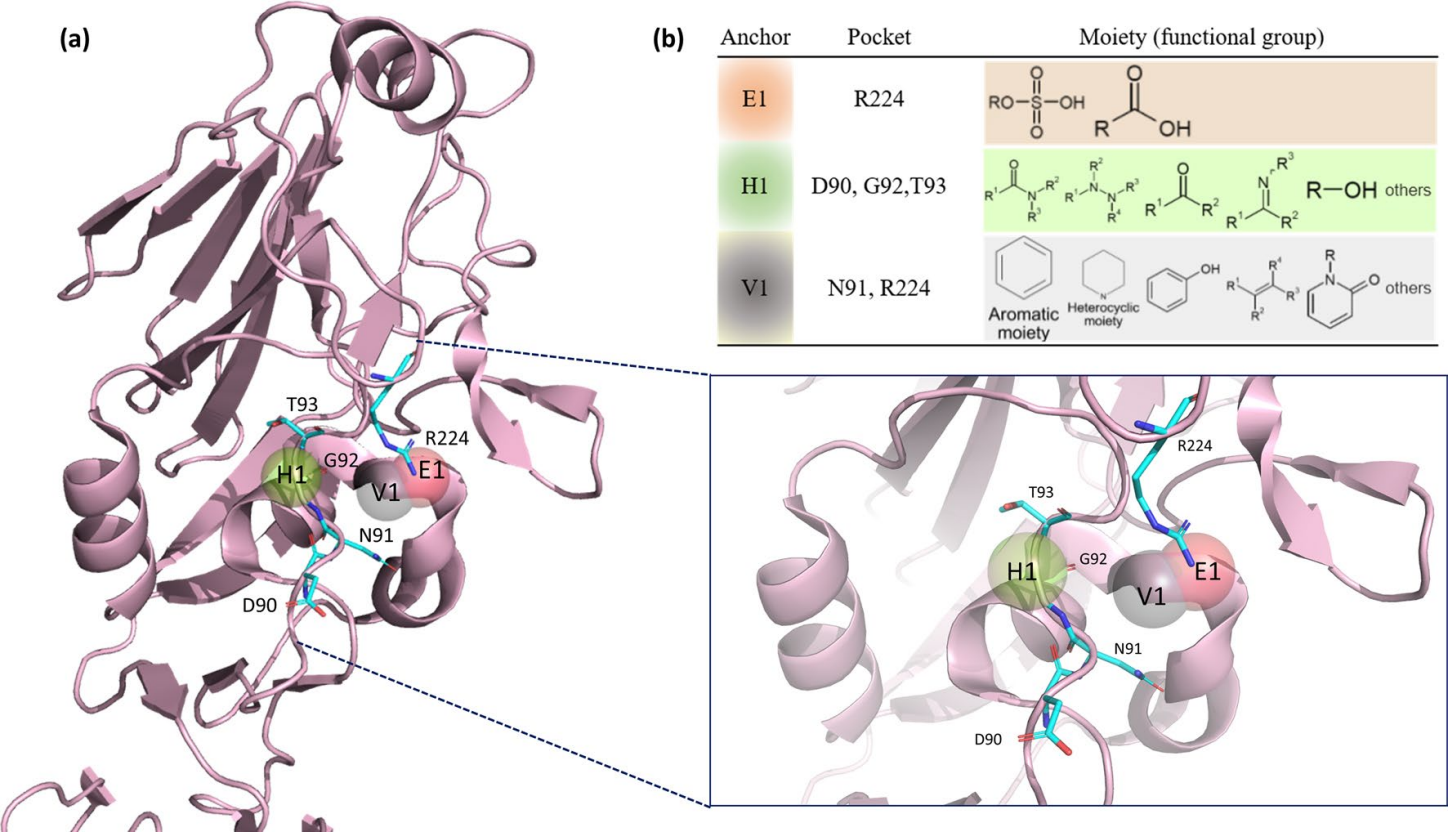
Receptor-binding site anchors obtained from SiMMap. **(a)** The RBS structure of the H1N1 HA (chain H of 1RUY) is depicted as a cartoon and three anchors as transparent spheres. Red E1 stands for the electrostatic force, green H1 for hydrogen bond force, and grey V1 for van der Waals forces. The corresponding binding pockets (residues) are shown in the cyan sticks. **(b)** The table presents binding pockets and moieties for each anchor. Each moiety of the anchor represents the functional group preference of the top-ranked compounds.

Three pivotal anchors, E1, H1, and V1 (Fig. 2b), were generated by SiMMap in the RBS. Of all three anchors and their respective pockets, sulfonic acid and carboxylic acid moieties prefer to interact with R224, the basic residue that lies in the 220-loop of the E1 anchor. Notably, all compounds that have a nucleophilic tendency, including sulfonic acid and carboxylic acid moieties, have a binding affinity with the E1 anchor. The H1 anchor interacts with one acidic residue (D90), a small residue (G92), and one nucleophilic residue (T93) located in the 90-loop. The H1 anchor prefers to bind to amide, ketone, amine, and hydroxyl groups (with decreasing preferences indicated in this order). Lastly, the V1 anchor has a high tendency to bind to an aromatic and heterocyclic moiety, interacting with one amide residue (N91) and one basic residue (R224), as well as the E1 anchor. Our 3-dimensional (3-D) model is shown in Fig. 2a.

### Compounds derived from NSC85561

**Figure 3:**
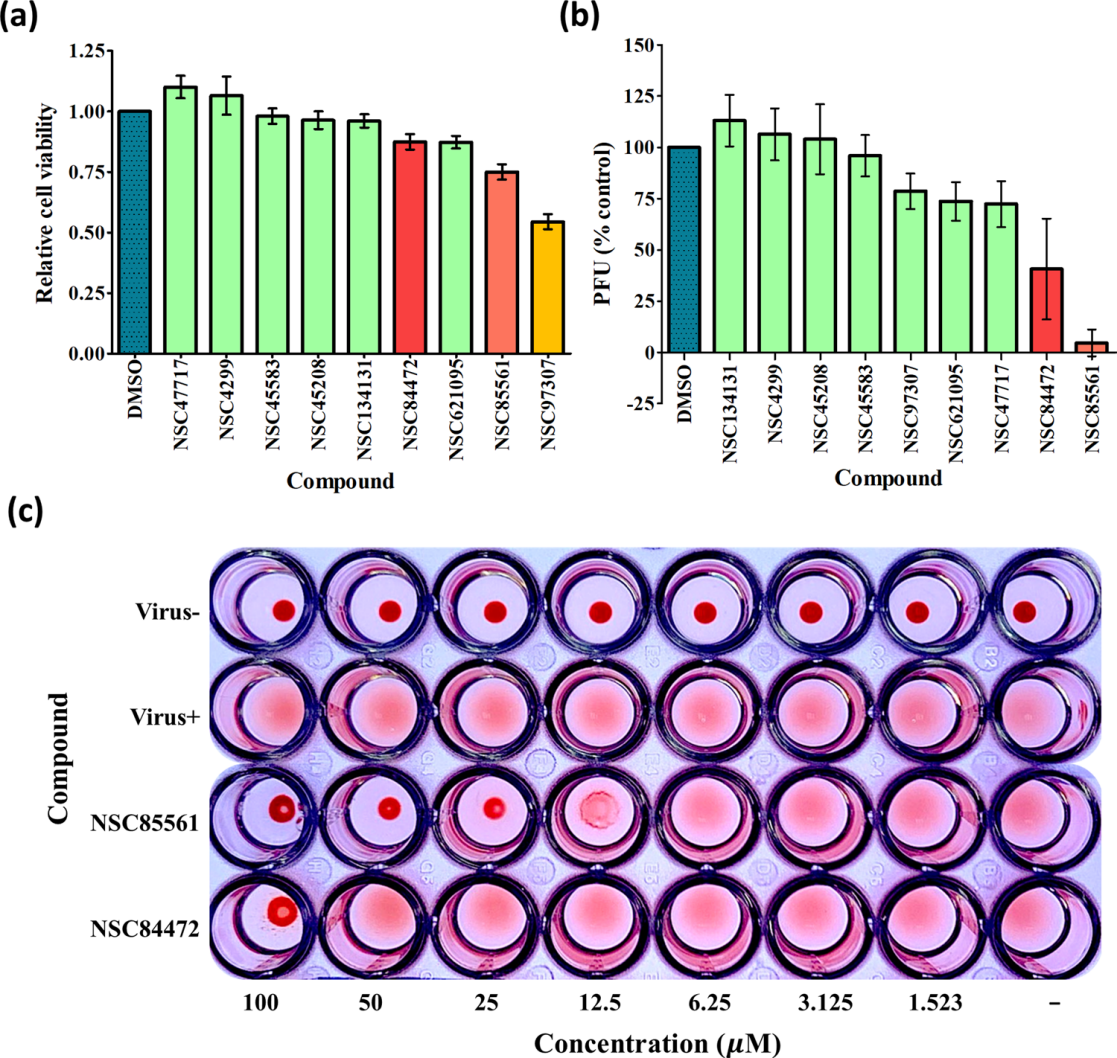
Identification of NSC85561 from the initial compounds. **(a)** MDCK cells were treated with the initial nine compounds at the concentration of 100 µM and cell viability was measured using the MTS assay. **(b)** MDCK cells were infected with A/WSN/1933 (H1N1, 100 PFU) were treated with the mixture at the concentration of 25 µM. A virus plaque reduction assay was performed. **(c)** Representation of the agglutination of NSC85561 and NSC84472 in serial two-fold dilutions started with 100 µM and was performed in three individual experiments.

Supplementary Table S1 lists the nine compounds that were purchased according to the SiMMap scores. The compounds were subjected to bioassay examinations and safety analysis using the MTS method^28^ (a proliferation assay that uses the tetrazolium compound, MTS, in combination with an electron coupling reagent to produce a colorimetric change). Each compound was applied as 100 µM concentrations to epithelial Madin Darby canine kidney (MDCK) cells to measure optical density (OD) values and toxic effects of the compounds (Fig. 3a). NSC97307 was excluded due to its low relative cell viability (below 0.75). When the cells were treated with 25 µM concentrations of each compound, identification of antiviral effects by plaque reduction assay revealed dramatic decreases from baseline in plaque-forming unit (PFU) values with NSC85561 and NSC84472 (mean PFUs of 5 ± 5% and 40 ± 20% versus control, respectively), identifying these compounds as effective inhibitors of the RBS (Fig. 3b and Table 1). Antiviral efficacy examination by HA assay began with concentrations of 100 µM for NSC85561 and NSC84472, followed by two-fold serial dilution. Whereas precipitation formed at 100 µM with NSC84472, precipitation did not occur with NSC85561 until the compound was diluted to 12.5 µM (Fig. 3c). Since the HA assay revealed much greater efficacy of NSC85561, we used this compound as a template to generate 12 further compounds based on the AtomPair fingerprints generated by RDKit^29^ Fingerprint in KNIME^30^. The AtomPair approach was used to generate 825 topological features^31,32^ for all of the compounds in our database, and these topological features were then used to measure the similarities between any two compounds by calculating the Pearson’s correlation coefficient (PCC). Any compound with a correlation coefficient greater than 0.8 was considered to be a derivative of the active compound NSC85561. The 2-D and PCC values of NSC85561 and the 12 derivative compounds are shown in Supplementary Table S2.

**Table 1:** Plaque reduction assay of the nine purchased compounds.

Compounds	PFU (% control)
NSC85561	5 ± 5
NSC84472	40 ± 20
NSC4299	106 ± 10
NSC47717	72 ± 9
NSC97307	79 ± 7
NSC621095	74 ± 8
NSC45583	96 ± 8
NSC45208	104 ± 14
NSC134131	113 ± 10

All experiments were performed in triplicate. Plaque-forming unit (PFU) values are shown as the mean ± standard deviation.

### Bioassay data matched the post-screening results

**Figure 4:**
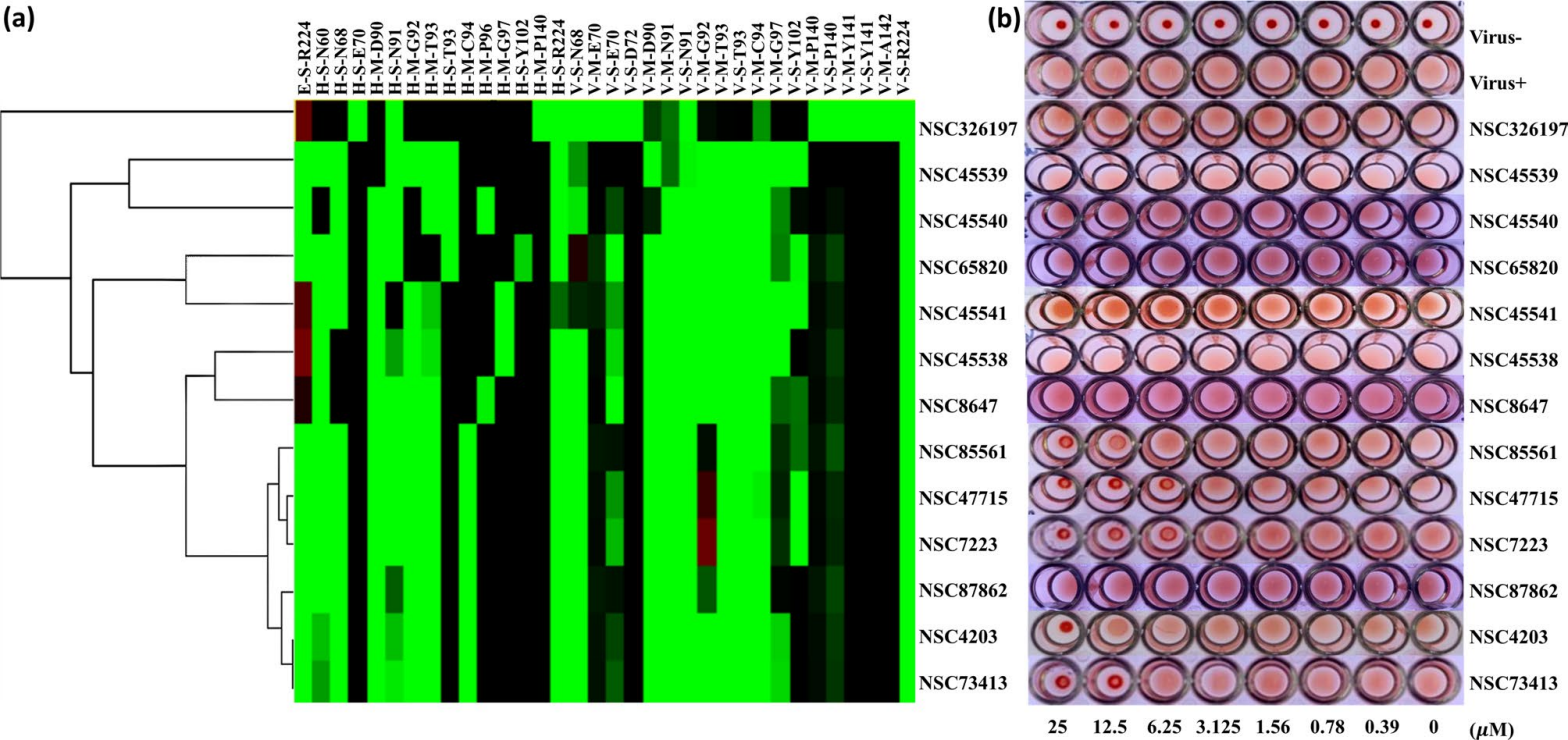
Similar outcomes from the post-screening analysis and HA inhibition assay. **(a)** The hierarchical clustering and interaction profiles of the 12 derivatives and NSC85561. The hierarchical tree represents compound similarities. The 13 compounds are listed on the y-axis and the interactive residues are listed on the x-axis. The first code of the interactive residue stands for the force between compounds and residues, E for electrostatic force, H for hydrogen bond force, and V for van der Waals forces. The second code stands for the interaction in the main chain (M) or side chain (S). The third code represents the residue type and serial number of the H1N1 HA. Residues with a pharmacological preference value of ≥ 0.4 are colored according to the interaction type: E = red; H = green; V = grey. The H or E interactions are represented in green when the energy ≤ –2.5. The V interactions are in green when the energy is < –4. **(b)** Representation of agglutination of the 13 compounds in serial two-fold dilutions, starting with 25 µM using the HA inhibition assay and performed in three individual experiments.

**Figure 5:**
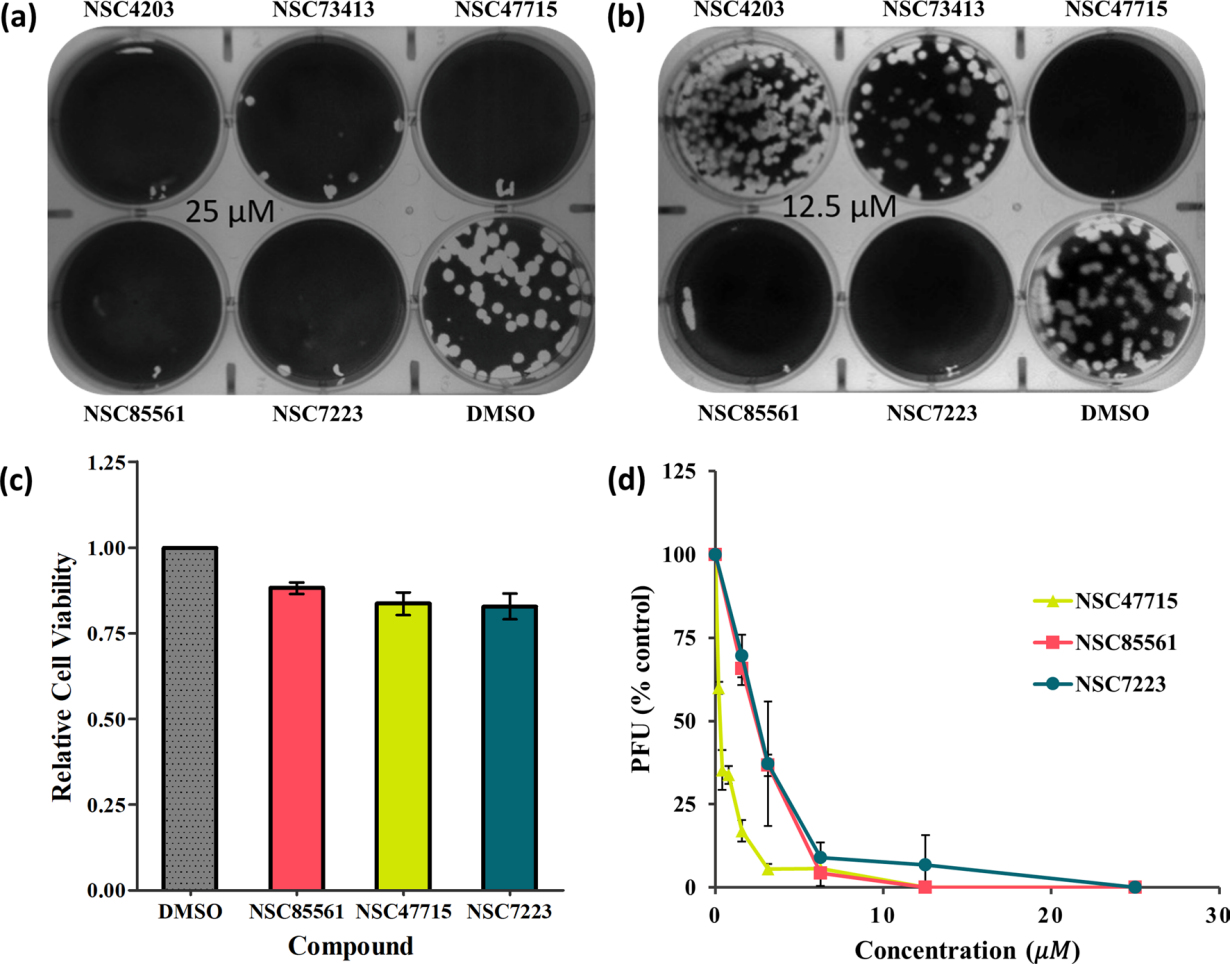
Identification of NSC47715, NSC85561 and NSC7223. MDCK cells were infected with A/WSN/1933 (H1N1, 100 PFU) and mixed with the five compounds at **(a)** 25 µM and **(b)** 12.5 µM. A virus plaque reduction assay was performed. **(c)** Relative cell viability value for NSC47715, NSC85561 and NSC7223 were evaluated by the MTS assay. **(d)** MDCK cells infected with the H1N1 virus were mixed with the three compounds in two-fold serial diluted concentrations. The plaque reduction assay was performed in at least three independent experiments.

The HA assay outcomes matched the iGEMDOCK post-screening results. Figure 4a depicts the hierarchical clustering results of 13 compounds according to their interaction profiles. Patterns were similar for 6 of the compounds (from NSC85561 to NSC73413), with evidence of early clustering (Fig. 4a). The HA experiments subjected all 12 derivative compounds and the initial compound to the two-fold serial dilution process, starting with a 25 µM concentration. Precipitation with NSC4203 formed at 25 µM, whereas this phenomenon occurred at 12.5 µM and over for both NSC85561 and NSC73413, and at ≥ 6.25 µM for both NSC47715 and NSC7223 (Fig. 4b). To validate the efficacies of these five compounds, we used different concentrations of the antiviral plaque reduction assay. The MDCK cells were seeded in 6-well plates for 24 h, then incubated with the five compounds at 25 µM or 12.5 µM (Fig. 5a,b). At the concentration of 12.5 µM, the efficacies of NSC47715, NSC85561, and NSC7223 were much higher than those of NSC4203 and NSC73413. Figure 5c illustrates the bar plot of the relative cell viabilities of NSC85561, NSC47715, and NSC7223. Average values were 0.88 for NSC85561, 0.83 for NSC47715, and 0.83 for NSC7223 at a concentration of 100 µM. The compound concentration required to reduce cell viability by 50% (CC_50_) was used to evaluate the safety of the three compounds. CC_50_ values were 700 µM for NSC85561, 900 µM for NSC7223, and > 1000 µM for NSC47715. The half-maximal concentration (EC_50_) was used to measure the efficacy of the three compounds. MDCK cells infected with the virus (100 PFU) were mixed with NSC47715, NSC85561, and NSC7223, and subjected to the virus plaque reduction assay (Fig. 5d). Among these three compounds, NSC47715 exhibited the lowest values, so was the most efficient at the lowest concentration at inhibiting IAV. Selectivity Index (SI) values (the CC_50_:EC_50_ ratio) evaluated cytotoxicity and efficacy. SI values were 289 for NSC85561, 360 for NSC7223, and > 3,000 for NSC47715. The above results are summarized in Table 2.

**Table 2:** CC_50 (µM)_, EC_50 (µM)_ and SI values for NSC47715, NSC85561 and NSC7223.

Compounds	CC_50_	EC_50_	SI	Score^a^	Energy^b^	VDW^b^	HBond^b^	Elec^b^	2-D structure
NSC47715	> 1000	0.29 ± 0.02	> 3448	3.233	–126.11	–72.98	–49.83	–3.30	
NSC85561	700	2.42 ± 0.11	289	3.228	–123.49	–69.96	–50.27	–3.25	
NSC7223	900	2.50 ± 0.76	360	3.227	–127.09	–71.48	–53.31	–2.30	

The EC_50_ value is presented as the mean ± standard deviation.

*CC*_*50*_ cytotoxic concentration 50%, *EC*_*50*_ half-maximal effective concentration, *VDW* van der Waals force, *Elec* electrostatic force.

^a^From SiMMap.

^b^From iGEMDOCK.

**Figure 6:**
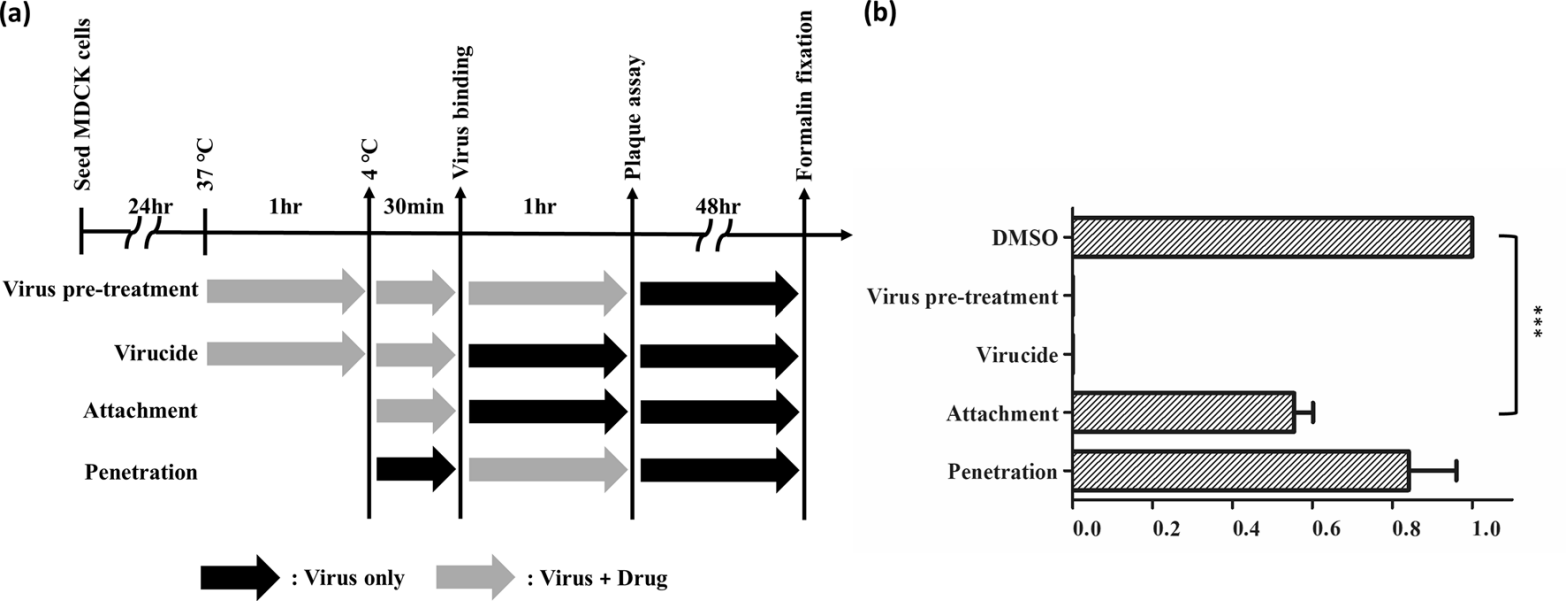
Time-of-addition assay. **(a)** Schematic representation of the antiviral activity of NSC47715 compound in different entry stages. **(b)** MDCK cells were infected with H1N1 (A/WSN/1933) and treated with NSC47715 compound at the concentration of EC_90(µM)_ at the indicated time according to the different strategy presented above. The values were presented as mean ± standard error of three independent experiments and were normalized to the values of DMSO solvent control. ***(*p* < 0.0001) shows there is a statistically different between NSC47715 and DMSO solvent control in the attachment stage.

Our previous data demonstrated that NSC47715 had the best potential in anti-IAV activity in the early stage of virus entry. Based on the results of the time-of-addition assay, NSC47715 was as expected to block the virus binding to the cells. However, the virucidal effect obviously contribute to the anti-IAV activity of the NSC47715. The virucidal effect represents the activity by which the compound interacts with virus and consequently prevents the binding of viral particles to the cells. Though the docking strategy is based on the blockage of the interaction of HA and sialic acid receptor, the compound may interact with HA causing the instability of virus structure leading to the failure binding to the cells. Taken together, NSC47715 displayed anti-IAV activity by blockage of virus binding to cells (Fig. 6b).

## Discussion

The genetic diversity of IAV poses an ongoing threat to global public health and much research has concentrated on the evolution of IAV and sought to clarify the mechanisms caused by the rapid mutations of the virus and genetic reassortment of gene segments^33^. The animal-human interface has also aroused the attention of scientists^34^ and encouraged the development of rigorous clinical guidelines for the prevention and treatment of influenza^35^. Few US FDA-licensed anti-influenza A virus drugs are available for clinical despite use, massive screening for new agents against IAV. The NA inhibitor oseltamivir is the main agent for use against IAV, but pathogen mutations have conferred resistance to oseltamivir in influenza A(H7N9) viruses^36,37^. Thus, NA inhibitors have limited efficacy against seasonal influenza viruses and emphasize the need to develop alternative anti-IAV therapies. Due to its critical role in the early stage of IAV, HA is a potential target protein for anti-influenza agents^38–40^. The HA sequence structure has been extensively explored and roughly divided into two parts: the SA receptor binding site and the fusion domain^25^.

In our study, the SA receptor binding site served as the target to block IAV infection, which differs from the recent research focusing on NA^41–43^. We first modeled the sialic acid RBS and built a compound dataset using NCI records. Using the process of molecule docking, we identified the top 1,000 compounds based on the iGEMDOCK energy values (Fig. 1a,b). To narrow down the list of compounds, we undertook post-virtual screening analysis on the SiMMap server to organize the orientation of the compounds and the HA residues, namely the anchors (Fig. 1c, Fig. 2). We examined the environment of anchors based on research published in 2019, in which the study researchers emphasized the vulnerable HA stalk trimer domain and depicted the conserved residues over various IAV strains^44^. They also found that FluA-20 (human antibody) tends to bind between the 220-loop and the 90-loop. Our models and predictions are consistent with their findings, so the anchors may play an essential role in interfering with the binding of IAV to the SA on the host cells.

Next, we used a bioassay to validate our prediction and hypothesis (Fig. 1d). NSC85561 was initially identified and served as the template to generate the derivatives from our compound database (Fig. 3). Supplementary Table S2 lists the PCC values lying between NSC85561 and the 12 derivatives generated from the atom-pair program. The PCC values range from 0.83 to 0.98, indicating similarity in the structure of the derivatives and the atom arrangement to those of NSC85561.

**Figure 7:**
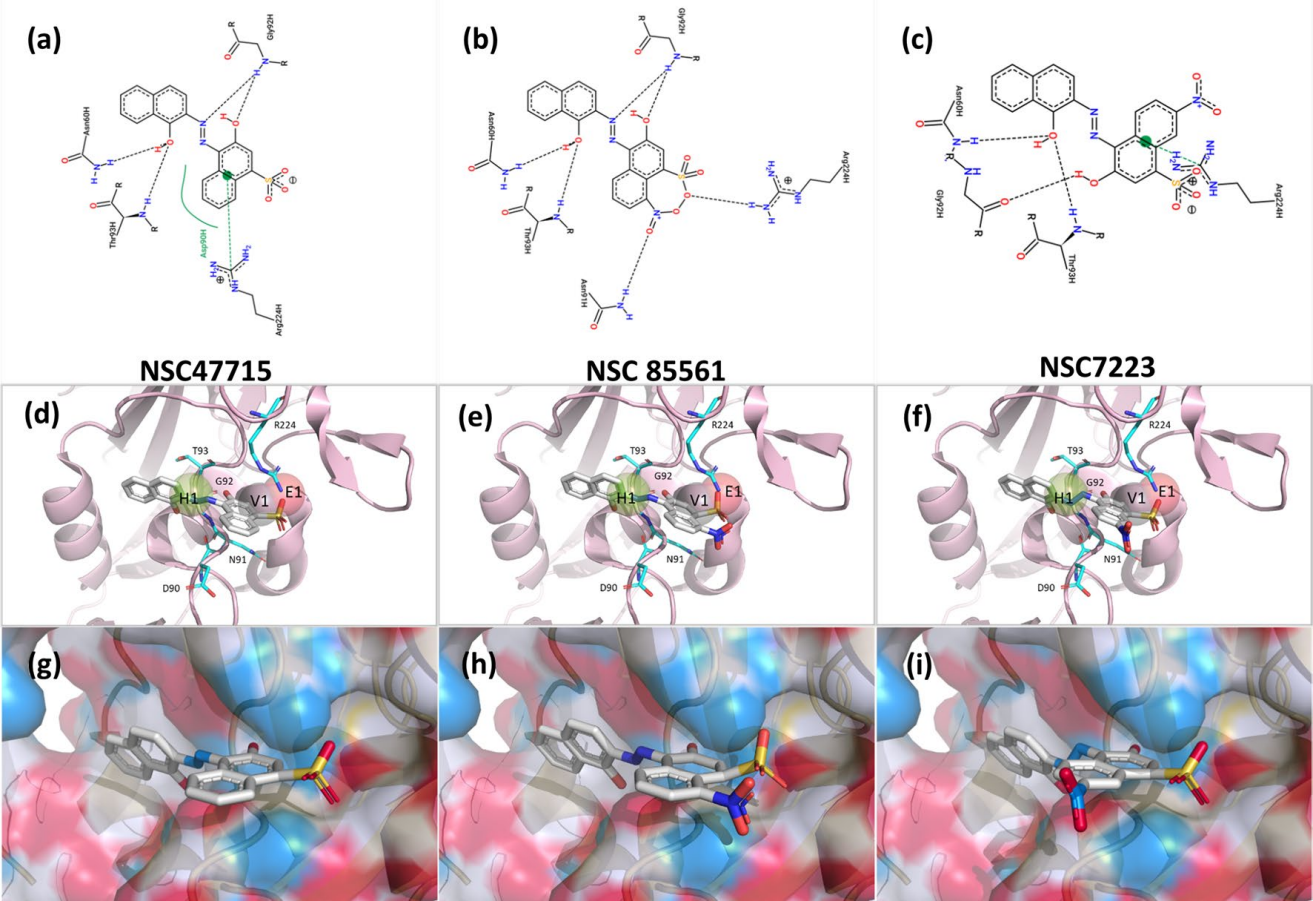
Docked compounds show a high affinity towards the RBS of the HA. The docked compounds **(a,d,g)** NSC47715, **(b,e,h)** NSC85561, and **(c,f,i)** NSC7223 are displayed in different visualization modes. **(a–c)** The interaction diagrams between docked compounds and proteins are shown in a 2-D plot that were examined by the PROTEINS PLUS server^45^. **(d–f)** Visualization of the docked compounds with anchors in the RBS. The HA structure is shown in the cartoon, anchors are shown as transparent spheres, interactive residues are shown as cyan sticks, and the docked compounds are shown as grey sticks. **(g–i)** The docked compounds in the RBS are represented in surface mode. PyMOL software was used to draw all of the 3-D figures^55^.

The interaction profile based on the post-screening analysis presents some intriguing outcomes (Fig. 4a). Although the patterns were similar for the six compounds from NSC85561 through to NSC73413, the anti-IAV efficacy of the HA assay was not as efficient with NSC87862 (Fig. 4b). Compared with the other five compounds, NSC87862 showed a lower binding affinity towards the side chain of the hydrogen-bonding force of N91 and the van der Waals force of Y102. Subsequently, the 2-D interaction diagrams of six compounds with the pocket environment were examined by the PROTEINS PLUS server^45^ (Fig. 7a–c and Fig. S1a–c), which revealed that all compounds except for NSC87862 had two naphthalene rings, which might explain their weak binding affinity. Moreover, the NSC4203 and NSC73413 naphthalene situated between residue T93 and G92 inhibits these compounds from fully binding with IAVs in that orientation, so NSC4203 and NSC73413 exhibit weak efficacy towards those IAVs (Fig. 7d–f and Fig. S1d-f). The RBS interface views of docked NSC47715, NSC85561 and NSC7223 is shown in Fig. 7g–i. Compared with NSC47715, the additional nitrite in NSC85561 and NSC7223 might interfere with their interaction with the HA binding site. As our investigations have shown, combining virtual screening and bioassay methods allows scientists to clearly and objectively identify novel SA binding site inhibitors.

## Conclusions

Our research sheds light on the feasibility of virtual screening generating SiMMap scores for identifying lead anti-IAV compounds. NSC85561 initially showed the highest affinity to the RBS, according to bioassay results. After conducting the same experimental assays on 12 derivatives of NSC85561, we identified three compounds, NSC85561, NSC7223, and NSC47715, as strong potential inhibitors of HA. The SiMMap server enables efficient searching for desired compound functional groups and elucidates the connection between the RBS and compounds NSC85561, NSC7223, and NSC47715. Thus, our data support the use of virtual screening methods to easily identify potential anti-IAV inhibitors that appear to exhibit much greater antiviral efficacy than existing strategies.

## Methods and materials

### Dataset preparation and virtual screening

The structure of H1N1 (PDB ID: 1RUY) was selected as the target protein for virtual screening from the Protein Data Bank^46^. H1N1 belongs to the H1 class from the 1930 swine influenza strain. Our preparation of the SA receptor binding site was as follows: Any residues located within 10 Å of the critical residues, K58, E70, S89, P140 and R224^47^, was selected as the SA receptor binding site/pocket in the 1RUY HA1 structure. To confirm whether or not our binding pocket is located in the conserved region of the HA1 subunit, the different hemagglutinin strains (all belonging to the H1N1 clade) were aligned using the multiple sequence alignment tool, Clustal Omega. The alignment results show that our prepared binding pocket is highly conserved in the HA1 subunit (Fig. S2). The 208,023 compounds employed for virtual screening were selected from the NCI database^48^ and were filtered out according to Lipinski’s Rule of Five. iGEMDOCK software^26^, a widely used docking program that provides accurate predictions, was used to dock the compounds into the RBS of the HA1 subunit. iGEMDOCK contains three types of interactions: electrostatic, hydrogen-bonding, and van der Waals forces. Based on the interactions (docking energy), which consist of a simple empirical scoring function and a pharmacophore-based scoring function, the top 1,000 compounds were selected.

### SiMMap construction and identification of hemagglutinin inhibitors

The top 1000 compounds ranked by docking energy with their binding poses were submitted to the SiMMap server for reevaluation^27^. The SiMMap was used to analyze a protein binding site to identify anchors (antigenic sites), in often critical binding environments^43^. Moiety compositions between these compounds and the SiMMap anchors provide clues for lead optimization. Based on the protein-compound interaction profiles generated by the SiMMap, consensus interactions between compound moieties and binding sites composed of consensus interacting residues were identified. Next, a site-moiety map score was generated for each chosen compound. The identified compounds were reordered with the scores. Finally, potential HA1 inhibitors were selected for rigorous examination based on their ranking, feasibility, physicochemical properties and interaction types.

### Cells and viruses

MDCK cells (ATCC accession no. NBL-2) were maintained in Dulbecco's modified eagle medium (DMEM; Gibco) containing 10% fetal bovine serum (FBS; Gibco), 1% penicillin and 1% streptomycin (PS; Gibco) at 37 °C in a 5% CO_2_ incubator. Influenza virus H1N1 (A/WSN/33) cells were supplied by Taiwan’s Centers for Disease Control and cultured in an influenza medium (DMEM with 1% PS and 2 μg/mL L-1-tosylamide-2-phenyl chloromethyl ketone [TPCK, Sigma-Aldrich]-Trypsin from bovine pancreas [Sigma-Aldrich, St. Louis, MO, USA]) for growth. All viruses were stored at –80 °C. All virus-related experiments were conducted in a BSL2 laboratory.

### Cell proliferation assay (MTS)

To test for cellular viability and cytotoxicity, we conducted a cell proliferation assay (MTS)^49^, a colorimetric method for sensitive quantification. Based on the reduction of MTS tetrazolium compound by viable cells, the MTS assay generates a colored formazan dye that can be quantified by measuring the absorbance at 490 nm. MDCK cells (1 × 10^4^ cells/well) were seeded into 96-well microtiter plates overnight then mixed with our potential compounds in influenza medium for 72 h at 37 °C under 5% CO_2_. The cells were washed twice with phosphate buffer saline (PBS; Na_2_HPO_4_ 8 mM, NaCl 137 mM, KCl 2.68 mM, KH_2_PO_4_ 1.47 mM, adjusted to pH 7.2), after the previous medium was removed from the wells. We then added 10 μL of MTS solution and 90 μL DMEM to each well, and the cells were incubated for 30 min. Finally, absorbance at 490 nm was recorded using a microplate reader (SpectraMax iD3, Molecular Devices, USA). A blank control (influenza medium only) and cell controls (without compounds defined as 100% cell survival) were included in every assay plate. The mean optical density (OD, absorbance) of the three wells in the indicated groups was used to calculate the percentage of cell viability as follows: Percentage of cell viability = (A_treatment_ − A_blank_)/(A_control_ − A_blank_) × 100% (A: absorbance)^50^. The cytotoxic concentration required for the identified compounds to reduce cell viability by 50% (CC_50_) was determined.

### Plaque assay

To determine viral titers as plaque-forming units per mL (PFU/mL), we selected the plaque assay^51,52^, which is a standard method for virus quantification. In brief, MDCK cells (1 × 10^6^ cells/well) were seeded in 6-well plates for 24 h at 37 °C under 5% CO_2_. A single layer of cells was infected with the indicated dose of IAV, followed by three washes with PBS. Next, the cells were covered with 0.3% agarose in the influenza medium for an additional two days at 37 °C. Cells were fixed with 10% formaldehyde and then stained with 1% crystal violet. Finally, the PFU was calculated.

### Plaque reduction assay

To quantify the antiviral activities of the compounds versus IAV, we conducted the virus plaque reduction assay. As described above, the previously seeded cellular monolayer (100 PFU/well) was incubated with or without the selected compounds. The virus suspension was removed following 1 h of IAV adsorption. The cells were immediately washed three times with PBS and overlaid with 0.3% agarose in the influenza medium, with or without the compounds, for 48 h. The EC_50_ values for all compounds were determined by comparing the number of plaques with those in the virus-infected control. The EC_50_ was defined as the half-maximal inhibitory concentration and the selectivity index (SI) was determined by the ratio of CC_50_/EC_50_.

### Time-of-addition assay

The time-of-addition (TOA) protocol determines how long the addition of a newly identified agent can be temporized before losing its antiviral ability during cell culture^53^. To understand the timing and stage at which the identified compounds inhibit IAV, the virus pretreatment protocol was divided into three stages (virucide, attachment, and penetration) to present the steps that virus may be blocked in the viral entry process^54^. The virucide stage: After hybridizing the drug and incubating the H1N1 (A/WSN/33) for an hour in a 6-well cell culture plate, we subjected the mixture to a 30-min rest at 4℃ before removing the mixture from the plate and subjecting it to incubation at 37℃, followed by the plaque reduction assay. In the attachment stage, the drug and virus were directly subjected to a 30-min rest at 4℃ in a cell culture plate, and then the mixture was removed, followed by the plaque reduction assay. In the penetration stage, the cell plate mixed with IAV was subjected to a 30-min rest at 4℃, then was added with the drug for another hour of incubation at 37℃, and then the mixture was removed, followed by the plaque reduction assay. This TOA approach is illustrated in Fig. 6.

### Hemagglutination inhibition assay

We used an erythrocyte agglutination test to determine whether SA bound to a compound and we examined whether the filtered compounds inhibited hemagglutination from causing a precipitation reaction in the SA binding and membrane fusion of HA. We diluted our compounds with PBS to 1:1,000. MDCK cells were diluted in a series of two-fold dilutions to achieve the IAV titer. To reduce the interference of the antibodies in erythrocytes, we prepared human type-O serum, which was diluted by two-fold serial dilutions in a 96-well microtiter plate.

## Supplementary Information

Supplementary Information.

## Data Availability

The authors confirm that the data supporting the findings of this study are available within the article and its supplementary materials.
