# Supplementary material for: Potent sialic acid inhibitors that target influenza A virus hemagglutinin
Source: Sci Rep. 2021 Apr 21;11:8637. doi: 10.1038/s41598-021-87845-0 (PMC8060387; doi:10.1038/s41598-021-87845-0)
Supplement: Supplementary file 1 — Supplementary Information. [file 41598_2021_87845_MOESM1_ESM.docx]

**Potent sialic acid inhibitors that target influenza A virus hemagglutinin**

Yu-Jen Chang^1,*^, Cheng-Yun Yeh^1,*^, Ju-Chien Cheng^2^, Yu-Qi Huang^2^, Kai-Cheng Hsu^3^, Yu-Feng Lin^4^ & Chih-Hao Lu^1,2,5^

^1^The Ph.D. Program of Biotechnology and Biomedical industry, China Medical University, Taichung, Taiwan

^2^Department of Medical Laboratory Science and Biotechnology, China Medical University, Taichung, Taiwan

^3^Graduate Institute of Cancer Biology and Drug Discovery, Taipei Medical University, Taipei, Taiwan

^4^Department of Medical Laboratory Science and Biotechnology, Asia University, Taichung, Taiwan

^5^Graduate Institute of Biomedical Sciences, China Medical University, Taichung, Taiwan

# *These authors contributed equally to this work.

# Correspondence

Chih-Hao Lu, The Ph.D. Program of Biotechnology and Biomedical industry, China Medical University, No.91, Hsueh-Shih Road, Taichung, 40402, Taiwan

Email: [chlu@mail.cmu.edu.tw](mailto:chlu@mail.cmu.edu.tw)

**Table S1.** The top 20 National Cancer Institute compounds identified from virtual screening ranked by SiMMap scores.

| **I-Rank** | **S-Rank** | **Compound** | **S-Score** | **E1** | **H1** | **V1** | **Purchased** | **2-D Structure** |
| --- | --- | --- | --- | --- | --- | --- | --- | --- |
| 1 | 1 | NSC97307 | 3.246 | 1 | 1 | 1 | V | 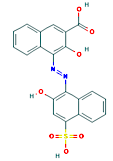 |
| 448 | 2 | NSC47717 | 3.207 | 1 | 1 | 1 | V | 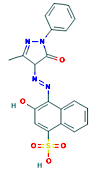 |
| 422 | 3 | NSC4299 | 3.205 | 1 | 1 | 1 | V | 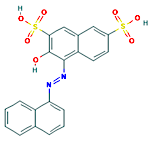 |
| 575 | 4 | NSC84472 | 3.205 | 1 | 1 | 1 | V | 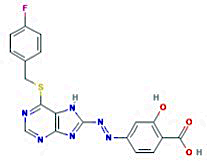 |
| 413 | 5 | NSC85561 | 3.205 | 1 | 1 | 1 | V | 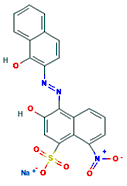 |
| 454 | 6 | NSC134131 | 3.201 | 1 | 1 | 1 | V | 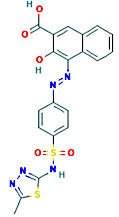 |
| 706 | 7 | NSC45583 | 3.199 | 1 | 1 | 1 | V | 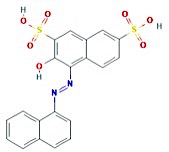 |
| 687 | 8 | NSC45208 | 3.196 | 1 | 1 | 1 | V | 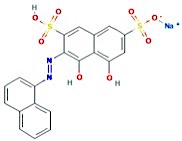 |
| 377 | 9 | NSC715307 | 3.196 | 1 | 1 | 1 |  | 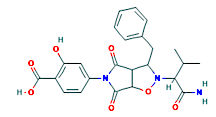 |
| 903 | 10 | NSC296652 | 3.193 | 1 | 1 | 1 |  | 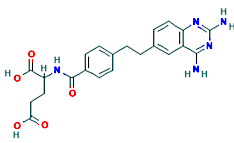 |
| 325 | 11 | NSC621095 | 3.192 | 1 | 1 | 1 | V | 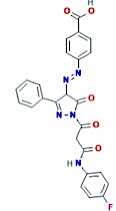 |
| 883 | 12 | NSC320860 | 2.273 | 0 | 1 | 1 |  | 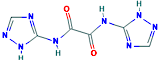 |
| 28 | 13 | NSC104528 | 2.27 | 0 | 1 | 1 |  | 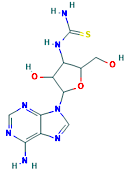 |
| 708 | 14 | NSC703071 | 2.269 | 0 | 1 | 1 |  | 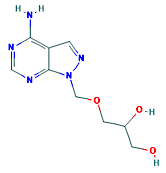 |
| 73 | 15 | NSC622187 | 2.267 | 0 | 1 | 1 |  | 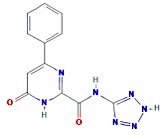 |
| 215 | 16 | NSC18193 | 2.263 | 0 | 1 | 1 |  | 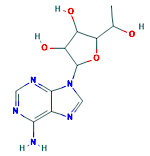 |
| 93 | 17 | NSC46715 | 2.259 | 0 | 1 | 1 |  | 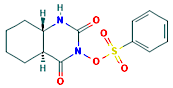 |
| 13 | 18 | NSC292236 | 2.258 | 0 | 1 | 1 |  | 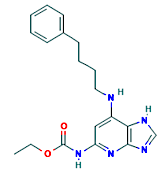 |
| 115 | 19 | NSC305183 | 2.257 | 0 | 1 | 1 |  | 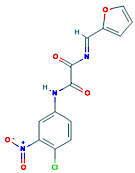 |
| 246 | 20 | NSC149033 | 2.255 | 0 | 1 | 1 |  | 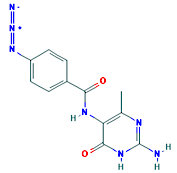 |
| Positive control | | Sialic acid | 3.216 | 1 | 1 | 1 |  | 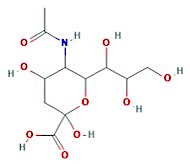 |

E1, H1, and V1 represent different anchors according to their force types. 1: Part of the compound is located in this anchor. 0: No part of the compound is situated in this anchor.

**Abbreviations:** I-Rank, iGEMDOCK rank; S-Rank, SiMMap rank; S-Score, SiMMap score; E1, electrostatic force; H1, hydrogen bond force; V1, van der Waals force.

**Table S2.** Structural and PCC values for NSC85561 and each of its 12 derivative compounds. The Pearson’s correlation coefficient (PCC), generated from the AtomPair program, describes the relationship between NSC85561 and each derivative. Two-dimensional structures are shown for NSC85561 and each of its 12 derivatives.

| **Compound** | **PCC** | **2-D Structure** |
| --- | --- | --- |
| NSC326197 | 0.83 | 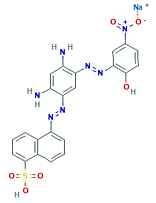 |
| NSC45539 | 0.9 | 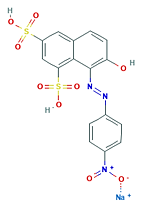 |
| NSC45540 | 0.89 | 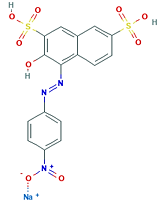 |
| NSC65820 | 0.90 | 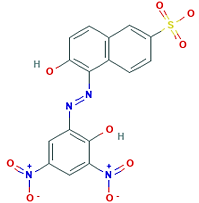 |
| NSC45541 | 0.88 | 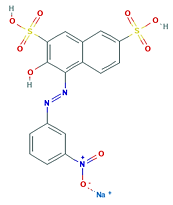 |
| NSC45538 | 0.94 | 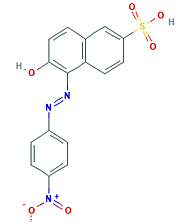 |
| NSC8647 | 0.90 | 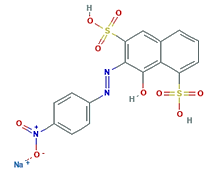 |
| NSC85561 | 1.00 | 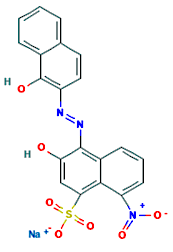 |
| NSC47715 | 0.96 | 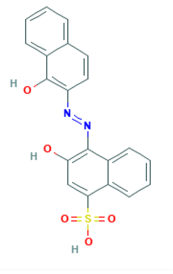 |
| NSC7223 | 0.98 | 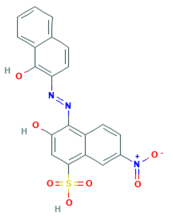 |
| NSC87862 | 0.91 | 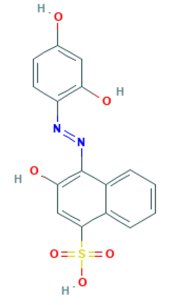 |
| NSC4203 | 0.95 | 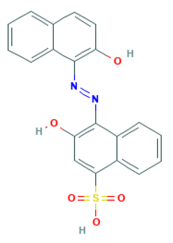 |
| NSC73413 | 0.98 | 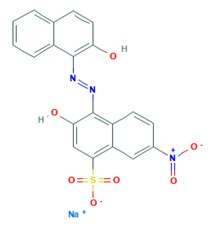 |

#
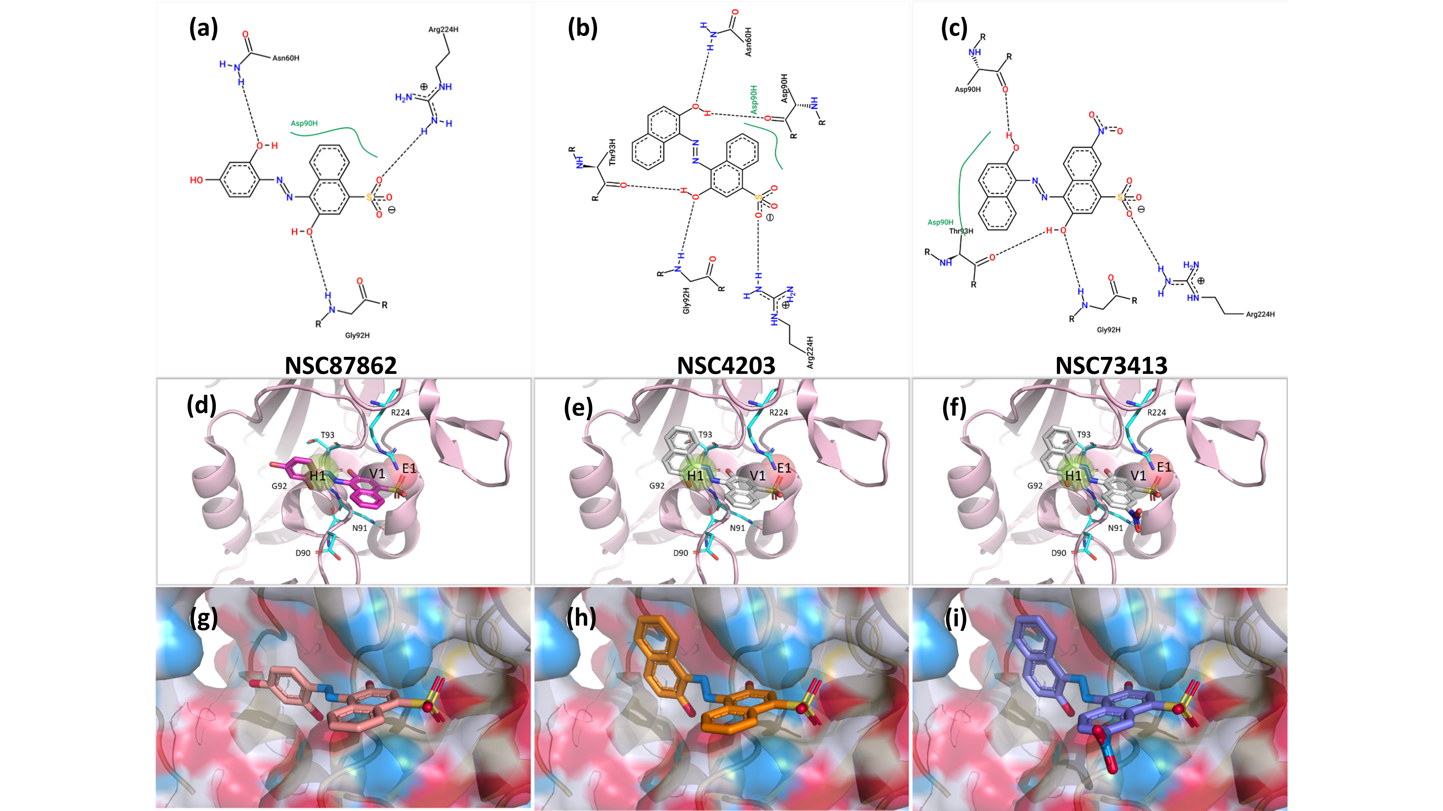


**Figure S1.** Docked compounds show a high affinity toward the RBS in HA.

The docked compounds **(a, d, g)** NSC87862, **(b, e, h)** NSC4203, and **(c, f, i)** NSC73413 are displayed in different visualization modes. **(a-c)** Interaction diagrams between the docked compounds and proteins are shown in 2-D plots that were examined by the PROTEINS PLUS server^1^. **(d-f)** Visualization of the docked compounds with anchors in the RBS. The HA structure is shown in the cartoon, with anchors represented as transparent spheres, interactive residues as cyan sticks, and the docked compounds as grey sticks. **(g-i)** The docked compounds in the RBS are represented in surface mode. PyMOL software was used to draw all of the 3-D figures^2^.


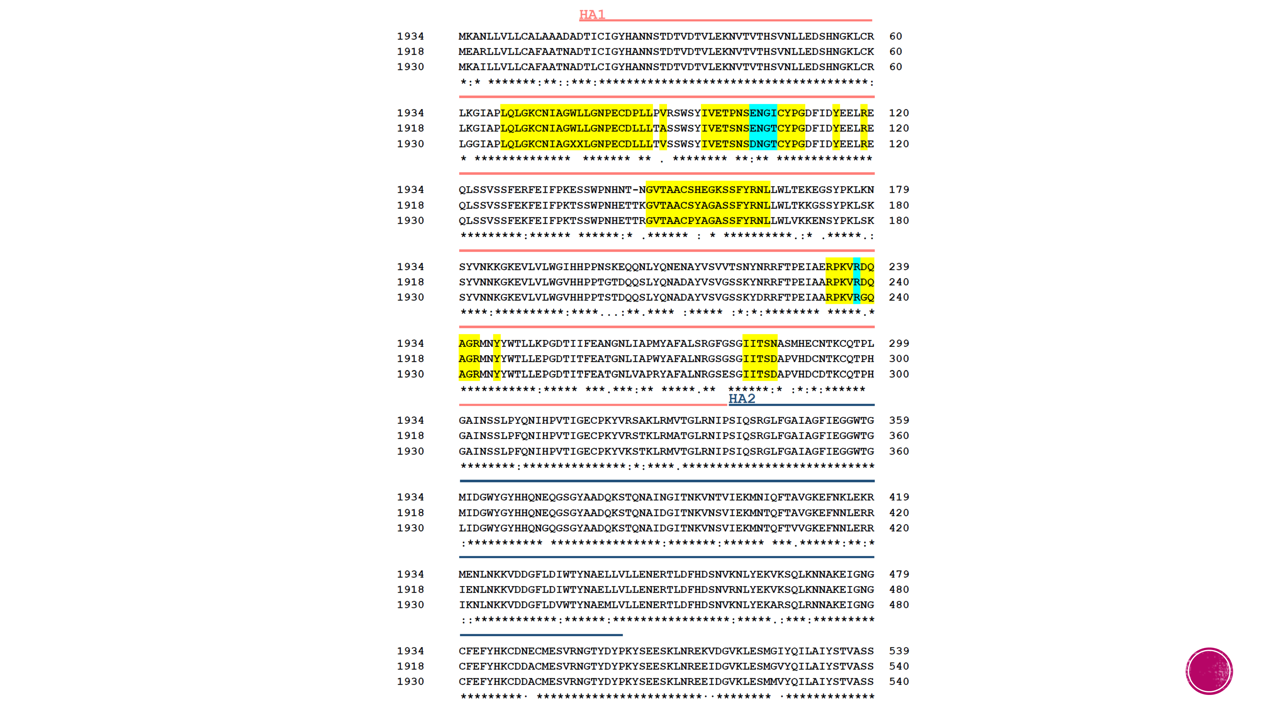


**Figure S2.** Sequence alignments for the 1918-human, 1930-swine, and 1934-human hemagglutinins. Colored bars above the sequences represent the HA1 and HA2 subunits, each of which forms a monomer. Residues of the sialic acid receptor binding sites are highlighted as yellow; critical anchor residues are highlighted in the color cyan. Residue numbers are on the right side of the figure.

1 Stierand, K., Maass, P. C. & Rarey, M. Molecular complexes at a glance: automated generation of two-dimensional complex diagrams. *Bioinformatics* **22**, 1710-1716, doi:10.1093/bioinformatics/btl150 (2006).

2 Schrödinger, L. The PyMOL Molecular Graphics System, Version 1.8. (2015).
